# A Fluorescence-Based Sensor Combined with Chemometric and Deep Learning Approaches for Detecting and Quantifying Coconut Milk Fraud in Bovine Milk

**DOI:** 10.3390/s26092872

**Published:** 2026-05-04

**Authors:** Stella Maria Dyah Cahyarani, Hoonsoo Lee

**Affiliations:** Department of Biosystems Engineering, College of Agriculture, Life, and Environment Sciences, Chungbuk National University, Cheongju 28644, Republic of Korea; stellamariadc02@chungbuk.ac.kr

**Keywords:** milk adulteration, excitation–emission matrix, fluorescence spectroscopy, deep learning, food authentication

## Abstract

Bovine milk adulteration with coconut milk poses a significant threat to food safety, as both liquids are visually similar yet nutritionally distinct. This study presents an integrated analytical framework combining excitation–emission matrix (EEM) fluorescence spectroscopy with chemometric and deep learning techniques to detect and quantify coconut milk adulteration in bovine milk across nine concentration levels (0–100% *v*/*v*). Parallel factor analysis (PARAFAC) resolved two dominant fluorescent components, tryptophan (λ ex/em: 290/350 nm) and riboflavin (λ ex/em: 450/525 nm), whose scores decreased monotonically with increasing adulteration, confirming their role as key chemical biomarkers. For quantitative prediction, PLSR and 1D-CNN models were developed using emission spectra at three excitation wavelengths, with best performances achieved at 450 nm (PLSR: R^2^P = 0.97, RMSEP = 5.00%; 1D-CNN: R^2^P = 0.94, RMSEP = 6.75%). A lightweight 2D-CNN utilizing full EEM contour maps as image inputs outperformed all quantitative models, achieving R^2^P = 0.99, RMSEP = 2.36%, and RPD = 12.97, demonstrating the advantage of preserving the full two-dimensional fluorescence topology over discrete wavelength selection. These results confirm that EEM combined with 2D-CNN provides a highly accurate and non-destructive tool for dairy authentication.

## 1. Introduction

Bovine milk is a global dietary agricultural commodity, important and valuable for human nutrition and recognized for its high nutritional value. It serves as a primary dietary source of essential macronutrients and micronutrients [1], providing proteins, carbohydrates, and fat; minerals and vitamins also contribute to its nutritional and biologic value [2]. Due to its high market demand, intensive production costs, and nutritional significance, bovine milk is consistently ranked among the most vulnerable targets for economically motivated adulteration (EMA) within the global food supply chain. EMA is defined as intentionally substituted or addition of a substance for the purpose of increasing the apparent value of the product or reducing the cost of its production [3]. To maximize profit margins, fraudulent actors frequently dilute pure milk with cheaper, visually similar alternatives.

In major tropical agricultural hubs such as Indonesia and Thailand, coconut milk (Cocos nucifera extract) is produced in massive quantities and costs much less than dairy farming, creating a highly profitable opportunity to illegally blend it into bovine milk. Because coconut milk is a plant-based white emulsion with a high lipid content, it perfectly mimics the macroscopic and organoleptic properties of cow milk [4]. However, it fundamentally lacks the specific dairy proteins and vitamins inherent to bovine milk [5], which negatively impacts the nutritional integrity of the adulterated product. Therefore, this form of fraud is virtually indistinguishable to the naked eye or through standard consumer evaluation, presenting a critical challenge for global food safety authorities and routine quality control.

Traditionally, the detection of food fraud relies on established chromatographic techniques, such as high-performance liquid chromatography (HPLC) or gas chromatography (GC). While highly accurate, these conventional methods are intrinsically limited for routine screening due to their high operational costs, time-consuming sample preparation, and extensive reliance on hazardous chemical solvents [6]. To overcome these logistical and environmental barriers, excitation–emission matrix (EEM) fluorescence spectroscopy has emerged as a highly effective alternative. EEM offers a non-destructive and entirely reagent-free approach to food quality assessment. This technique is exceptionally sensitive to endogenous fluorophores present in complex biological mixtures [7]. In the context of dairy analysis, bovine milk exhibits strong, distinct fluorescence signals driven primarily by the amino acid tryptophan within its protein structure, alongside intrinsic vitamins like riboflavin [8]. In contrast, coconut milk introduces a completely different biochemical profile, lacking these specific fluorophores. By capturing the complete multi-dimensional spectral landscape of these mixtures, EEM provides a highly sensitive fingerprint.

Despite its analytical advantages, EEM generates massive and highly complex three-dimensional (3D) matrices. In not-clear liquids like milk emulsions, these datasets are further complicated by intensely overlapping fluorophores and severe light scattering effects [9]. Usually, chemometric techniques are employed to explain these signals. Parallel factor analysis (PARAFAC) is widely utilized to resolve hidden chemical components [10] providing a robust basis for discriminating authentic samples from adulterated ones. To perform quantitative analysis and determine the precise percentage of the adulterant, partial least squares regression (PLSR) remains the conventional standard. However, PLSR possesses a critical mathematical limitation: it necessitates flattening the 2D excitation–emission matrix into a one-dimensional (1D) array prior to modeling. This forced transformation inherently destroys the natural spatial relationships and topographical integrity of the fluorescence landscape, frequently resulting in compromised predictive accuracy when evaluating highly intricate, non-linear emulsion mixtures.

Recent studies have highlighted the growing potential of combining spectroscopy with machine learning for food adulteration analysis and intelligent analytical sensing: for example, [11] applied FTIR spectroscopy with LDA-based feature extraction and KNN classification to detect adulteration in coconut milk, reporting a cross-validation accuracy of 93.33%. A broader food-analysis context [12] showed that spectroscopy coupled with an extreme learning machine can provide effective food classification performance, demonstrating the utility of advanced machine-learning algorithms for spectroscopic data interpretation. In addition, [13] developed a machine-learning-assisted fiber-optic Raman sensor for rapid, real-time detection of foodborne pathogens, further illustrating the analytical value of integrating spectroscopy with data-driven sensing strategies. However, most previous studies have focused mainly on one-dimensional vibrational spectra and classification-oriented tasks, while quantitative modeling of complex fluorescence matrices in emulsion-based adulteration systems remains limited.

Building on these advances, recent studies have widely adopted deep learning algorithms to construct advanced classification and regression models for high-dimensional spectral datasets. Convolutional neural networks (CNN) have emerged as a powerful tool in food authentication [14,15]. For example, in milk analysis, a CNN-based model achieved an accuracy of 97.7% [16]. Beyond standard CNN architectures, more complex deep learning models such as ResNet with residual connections [17] and Transformer-based architectures with self-attention mechanisms have also demonstrated strong performance in spectroscopic food analysis [18]. Because the CNN process inputs as multi-dimensional arrays, it is uniquely suited to evaluate two-dimensional spectral data by treating it as an image. Through the application of localized convolutional filters, CNNs can automatically extract hierarchical spatial features, capturing both subtle intensity gradients and complex non-linear relationships directly from the spectral landscape [19]. To overcome the spatial degradation caused by traditional PLSR, this study proposes an image-based analytical approach utilizing a customized deep learning architecture. By converting normalized EEM data into visual contour maps (2D RGB images), CNN can directly interpret the intact fluorescence topography [20]. However, given the small dataset size (*n* = 90) inherent to this study, a lightweight single-layer 2D-CNN was deliberately chosen to balance model expressivity with overfitting risk, a design philosophy supported by recent studies demonstrating that architecturally simple CNNs are competitive with deeper networks on limited spectroscopic datasets [21]. Despite its structural simplicity, this streamlined model is hypothesized to effectively capture the nuanced spatial features of the EEM matrices, thereby delivering superior quantitative performance over conventional chemometric techniques.

Therefore, the primary objective of this study is to establish an accurate and advanced analytical framework for detecting coconut milk adulteration in bovine milk using EEM. Specifically, this research aims to: (1) resolve the underlying chemical fluorescence profiles of pure and blended mixtures using PARAFAC; (2) evaluate the quantitative predictive performance of the traditional chemometric approach (PLSR) using emission spectra at selected excitation wavelengths; (3) assess whether a deep learning approach using identical one-dimensional spectral inputs (1D-CNN) can improve upon PLSR performance; and (4) investigate whether a lightweight image-based 2D-CNN utilizing the full EEM contour map as input can further enhance predictive accuracy by preserving the complete two-dimensional fluorescence topology. The predictive robustness of these models is systematically evaluated using standard analytical metrics, including the coefficient of determination (R2), root mean square error (RMSE), ratio of performance to deviation (RPD), and limit of detection (LOD). Through this approach, this study seeks to provide a highly efficient, reagent-free screening tool for food safety authorities to effectively prevent economically motivated adulteration in the global dairy supply chain.

## 2. Materials and Methods

### 2.1. Sample Preparation

In this study, two commercial brands of cow milk were purchased from local retail outlets, while one brand of coconut milk was selected as the adulterant. To maintain sample integrity and prevent microbial degradation, all samples were stored at 5 °C in a refrigerated laboratory chamber prior to analysis. The experimental groups were categorized into nine concentration levels of coconut milk: 0% (pure cow milk), 2.5%, 5%, 10%, 20%, 30%, 40%, 50%, and 100% (pure coconut milk). For each concentration, 10 technical replicates were prepared using the same source products. To minimize batch-specific preparation bias, these replicates were not drawn from a single large batch. Instead, each replicate was prepared individually from scratch and thoroughly homogenized using a vortex mixer immediately prior to scanning. This resulted in a total of 90 spectral scans for data analysis.

### 2.2. Instrument

**Figure 1:**
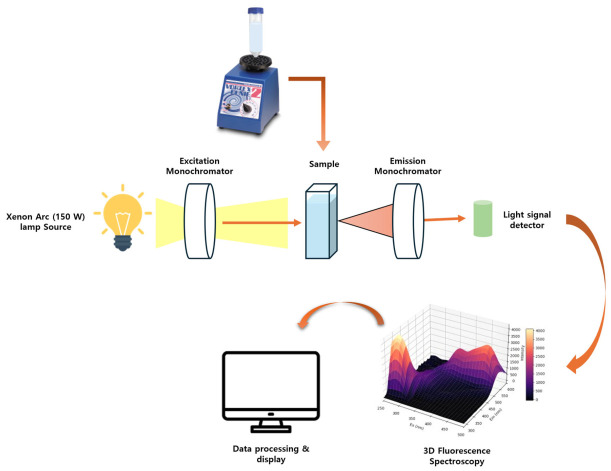
Schematic diagram of the instrumental setup for excitation–emission matrix (EEM) fluorescence data acquisition.

The fluorescence spectra of pure milk and adulterated milk samples were measured by FL8500 fluorescence spectrophotometer (PerkinElmer, Waltham, MA, USA). The schematic diagram of the instrumental setup and data acquisition process is illustrated in Figure 1. Excitation wavelengths were scanned from 250 to 500 nm with a 5 nm step interval, while emission wavelengths ranged from 280 to 600 nm. These specific spectral ranges were strategically selected based on established literature to encompass the excitation and emission maximum of the primary intrinsic fluorophores present in dairy products, such as tryptophan, vitamin A, and riboflavin [8]. The slit width was set to 5 nm for both excitation and emission channels and the scan speed was 240 nm/min.

### 2.3. Data Preprocessing and Scattering Removal

Raw fluorescence excitation–emission matrix (EEM) data inherently contains non-fluorescent scattering, primarily the first- and second-order Rayleigh and Raman scatterings [22,23], which can obscure true chemical fluorophore signals and interfere with subsequent multivariate modeling [24]. Therefore, a mathematical preprocessing protocol was implemented prior to any chemometric or deep learning analysis.

Initially, the specific regions affected by these scattering were mathematically masked from the raw EEM landscapes. The first order scattering region was removed within a bandwidth of ± 25 nm around the exact excitation, while the second order Rayleigh scattering was excised within a bandwidth of ± 20 nm around twice the excitation wavelengths [25,26]. To reconstruct the missing data within these excised regions, a two-dimensional cubic interpolation algorithm was applied across the excitation and emission grid [27]. This ensured a continuous and seamless topographical transition between the scattering boundaries and the fluorescence peaks.

Furthermore, a Gaussian filter with a standard deviation (σ) of 1.5 was applied to the interpolated EEM matrices [28]. To smooth the data, any resulting negative values were removed numerically to maintain the physical validity of the fluorescence intensity [29]. These fully cleaned matrices were subsequently used for PARAFAC, PLSR, 1D-CNN, and 2D-CNN analyses.

### 2.4. Parallel Factor Analysis (PARAFAC)

To resolve the overlapping fluorescence signals inherently present in the emulsion matrices, the preprocessed three-way EEM data array was subjected to parallel factor analysis (PARAFAC) [30]. The trilinear decomposition is mathematically expressed as [24,31]:

(1) $$x_{i,j,k}=\sum_{f=1}^{F}a_{if}b_{jf}c_{kf}+e_{ijk}$$

In Equation (1), $x_{i,j,k}$ correspond to fluorescence intensity, $a_{if}$, $b_{jf}$, $c_{kf}$ are the relative concentration emission and excitation loadings for the f-th component, and $e_{ijk}$ is the residual error. Consequently, these loadings and scores serve as reliable estimates of the true chemical fluorophores within the samples.

The PARAFAC modeling was performed using the TensorLy library in Python. For computational stability, the previously excised Rayleigh scatter regions were numerically replaced with zeros prior to the decomposition [32]. Since physical fluorescence intensity cannot be negative, strict non-negativity constraints were applied across all three modes [25,33]. The algorithm was initialized using singular value decomposition (SVD) with a convergence tolerance of 1 × 10^−6^ and a maximum limit of 2000 iterations. Finally, a two-component model was selected based on the logical extraction of the non-negative spectral profiles and their corresponding concentration scores.

### 2.5. PLSR

To quantify the level of adulteration in coconut milk, partial least squares regression (PLSR) models were developed. Based on the primary chemical fluorophores identified during the PARAFAC analysis, specific excitation wavelengths were extracted from the 2D EEM matrices to form 1D emission spectra. Prior to modeling, the dataset was randomly partitioned into a calibration set (70%) for model training and an independent prediction set (30%) for model validation.

A data augmentation strategy based on the method proposed by [34] was applied to the calibration set to improve model robustness and prevent overfitting. This technique expanded the calibration data by a factor of nine by introducing random background offsets, multiplicative scaling, and slope variations. The prediction set remained unaugmented to ensure a highly objective evaluation of the model true predictive capability.

To evaluate how light scattering affects the model’s accuracy, the models were developed using raw (unprocessed) spectra as a baseline and compared with spectra preprocessed using multiplicative scatter correction (MSC) and Savitzky–Golay first derivative (SG1). Finally, the model performance was evaluated using the coefficient of determination (R^2^) and the root mean square error (RMSE) for both the calibration and prediction sets, along with the residual predictive deviation (RPD) to confirm its practical reliability.

### 2.6. One-Dimensional Convolutional Neural Network (1D-CNN) Modelling

To evaluate the efficacy of deep learning for spectral feature extraction on isolated emission vectors, and to serve as an algorithmic bridge between linear PLSR and spatial 2D-CNN, a lightweight 1D-CNN was developed. The models were independently constructed and evaluated for the three diagnostic excitation wavelengths identified via PARAFAC analysis (290 nm, 330 nm, and 450 nm).

#### 2.6.1. Data Preprocessing

For each targeted excitation wavelength, the corresponding one-dimensional emission spectra was extracted from the cleaned EEM matrix of each sample. To ensure a robust evaluation, the dataset (*n* = 90) was partitioned into a calibration set (70%) and an independent prediction set (30%) using a stratified splitting strategy based on the adulteration concentration levels. To mitigate the risk of model overfitting associated with limited sample sizes in deep learning, a spectral data augmentation strategy was applied to the calibration set. Consistent with the PLSR methodology, the original calibration spectra were expanded by a factor of 9. This was achieved by introducing randomized variations (background noise, multiplicative scaling, and baseline slope shifts) to simulate real-world instrumental fluctuations. Furthermore, z-score standardization was applied to normalize the spectral intensities. The finalized input tensors were reshaped to accommodate the 1D-CNN channel requirements.

#### 2.6.2. 1D-CNN Architecture

**Figure 2:**
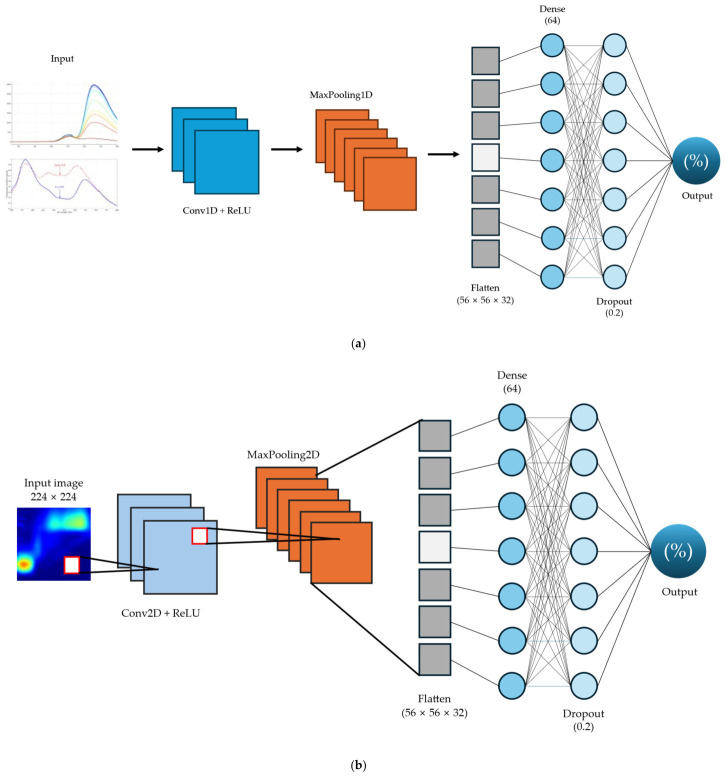
Deep learning architecture (**a**) 1D-CNN; (**b**) 2D-CNN.

The architecture consisted of one convolutional block for feature extraction, presented in Figure 2a. The block utilized a 1D convolutional layer with 16 filters (kernel size of 3, HeNormal initializer), followed by a ReLU activation function and a 1D max-pooling layer (pool size of 2). Following feature extraction, the feature maps were flattened and fed into a fully connected layer with 64 neurons (ReLU activation), protected by a dropout layer with a rate of 0.2. The final output layer used a linear activation function to predict adulterant concentration.

The model was compiled using the Adam optimizer with an initial learning rate of 0.001. The loss function was defined as mean squared error (MSE) and mean absolute error (MAE) was monitored as an additional metric. Training was executed for a maximum of 300 epochs using a batch size of 16. To ensure optimal model generalization and prevent overtraining, an early stopping callback was implemented to halt training if the validation loss did not improve for 20 consecutive epochs, automatically restoring the best model weights. Additionally, a learning rate scheduler (ReduceLROnPlateau) was applied to dynamically reduce the learning rate by a factor of 0.5 if the validation loss plateaued for 10 epochs, down to a minimum learning rate of 10^−6^. This 1D-CNN was implemented in Python using the Keras API within the TensorFlow framework.

### 2.7. Two-Dimensional Convolutional Neural Network (2D-CNN) Modelling

#### 2.7.1. Image Preprocessing (Normalization and Augmentation Process for 2D-CNN)

To prepare the raw fluorescence spectra for the 2D-CNN architecture, a systematic preprocessing pipeline was implemented. Initially, the raw excitation–emission matrices (EEMs) were subjected to global maximum normalization to ensure uniform intensity scaling and prevent signal distortion across different samples [35,36]. The normalization process is mathematically defined as follows:

(2) $$X_{norm}=\frac{X_{raw}}{X_{global\_max}}\times 255$$

where $X_{norm}$ is the normalized matrix scaled to an 8-bit integer range (0–255), $X_{raw}$ is the original instrument-derived fluorescence intensity matrix, and $X_{global\_max}$ represents the absolute maximum fluorescence intensity recorded across the entire dataset. As demonstrated by [37], the Convolutional Neural Network (CNN) inherently performs better with image-based data. This conversion approach effectively preserves the spatial and topological relationships of the fluorophores before being input into the proposed 2D-CNN regression model. To standardize the inputs, the resulting contour maps initially saved at a resolution of 128 dpi with physical dimensions of (4 × 4) inches, yielding an intermediate image size of (512 × 512) pixels.

To ensure an unbiased evaluation, the dataset was divided into a training set (70%) and an independent test set (30%) prior to any further processing. Following this data split, a mathematical data augmentation strategy was applied to the training set to expand the sample size and mitigate the risk of overfitting. Previous studies, by [38,39], have demonstrated that introducing artificial noise during the training phase significantly enhances the generalization capabilities of neural networks. Therefore, a hybrid augmentation strategy combining amplitude scaling and noise injection was implemented. Both techniques are well established for reducing overfitting and improving the robustness of predictive models [40]. Inspired by the affine transformation principles described by [37], amplitude scaling using factors ranging from 0.85 to 1.15 was applied. This amplitude variation step is crucial to represent changes in the equipment sensitivity under varying operating conditions and simulate natural scattering fluctuations. Furthermore, random Gaussian noise (with standard deviations ranging from 1.0 to 5.0) was injected into the augmented spectra to simulate dark current detector and thermal noise [41], which is expressed by the following formula:

(3) $$X_{m\;\times \;n}^{noise}=X_{m\;\times \;n}+N\;(0,\sigma )$$

where $X_{m\;\times \;n}^{noise}$ and $X_{m\;\times \;n}$ are the fluorescence intensity matrices with and without noise, respectively. The function N (0,  σ) generated a noise matrix of the same dimensions as the input data. This approach ensures the 2D-CNN learns robust chemical features, while the test set remains completely unaugmented to provide an objective prediction of the regression model.

#### 2.7.2. 2D-CNN Architecture

To automatically extract complex spatial fluorescence features without relying on manual mathematical preprocessing, a custom 2D-CNN was developed for regression analysis, as illustrated in Figure 2b. The deep-learning framework utilized in this work was inspired by the structural principles of EEM image-based regression proposed by [42,43]. The preprocessed and augmented EEM contour matrices were mapped into 2D images and resized to 224 × 224 pixels with three color channels (RGB). Prior to network ingestion all image pixel values were normalized to a range of 0 to 1 to ensure computational stability.

The architecture consisted of one convolutional block for feature extraction. The block utilized a 2D convolutional layer with 16 filters (3 × 3 kernel, HeNormal initializer), followed by a ReLU activation function and a max-pooling layer (2 × 2 pool size). Following feature extraction, the feature maps were flattened and fed into a fully connected layer with 64 neurons (ReLU activation), protected by a dropout layer with a rate of 0.2. The final output layer used a linear activation function to predict adulterant concentration.

The model was compiled using the Adam optimizer with an initial learning rate of 0.0005. The loss function was defined as mean squared error (MSE), and mean absolute error (MAE) was monitored as an additional metric. Training was executed for a maximum of 300 epochs using a batch size of 16. To ensure optimal model generalization and prevent overtraining, an early stopping callback was implemented to halt training if the validation loss did not improve for 20 consecutive epochs, automatically restoring the best model weights. Additionally, a learning rate scheduler (ReduceLROnPlateau) was applied to dynamically reduce the learning rate by a factor of 0.5 if the validation loss plateaued for 10 epochs, down to a minimum learning rate of 10^−6^. This 2D-CNN was implemented in Python using the Keras API within the TensorFlow framework.

To evaluate the specific impact of the data augmentation strategy [34] on model performance, two separate 2D-CNN models were developed and compared. It is crucial to note that both models utilized the identical 2D-CNN architecture (consisting of one convolutional block, flattening, and dense layers) and the same training parameters defined above. The only difference between the two models was the composition of the input training data. The first model, referred to as the non-augmented CNN, was trained using only the limited original calibration dataset (*n* = 63 samples). The second model, referred to as the augmented CNN, was trained using the expanded calibration dataset (*n* = 630 samples) generated via the augmentation technique. Both models were validated using the same independent prediction set (*n* = 27 samples) to ensure a fair and objective comparative analysis.

### 2.8. Statistical Analysis

The performance of the PLSR, 1D-CNN, and 2D-CNN models was evaluated by comparing the predicted coconut milk adulteration level against the reference concentration values. The accuracy and robustness of the models were assessed using the coefficient of determination (R^2^), the root mean square error (RMSE), the residual predictive deviation (RPD), and the limit of detection (LOD). In this study, RMSEV was used to represent the model error during the validation stage, whereas RMSEP was used to evaluate the predictive performance of the final model on the independent prediction set. Therefore, both RMSEV and RMSEP are reported, as they describe different aspects of model performance: RMSEV reflects internal validation error, while RMSEP indicates the practical prediction accuracy of the final model. Since the LOD was estimated using the multivariate definition based on validation error, it was calculated as Equation (6), following the multivariate LOD definition proposed by [44]. An optimal model is characterized by high R^2^ and RPD values coupled with a low RMSE and LOD. The mathematical formulas for these parameters are presented in Equations (4)–(6) [44,45].

(4) $$R^{2}=1-\frac{\sum_{i=1}^{n}{\left(y_{i,p}-y_{i,a}\right)}^{2}}{\sum_{i=1}^{n}{\left(\bar{y}-y_{i,p}\right)}^{2}}$$

(5) $$RMSE=\sqrt{\frac{\sum_{i=1}^{n}{\left(y_{i,p}-y_{i,a}\right)}^{2}}{n}}$$

(6) LOD=3×RMSEV

### 2.9. Software

All analyses were implemented using Python (version 3.13.5) scripted in Visual Studio Code (version 1.107.0; Microsoft Corporation, Redmond, WA, USA) software.

## 3. Results and Discussion

### 3.1. Spectral Characterization

**Figure 3:**
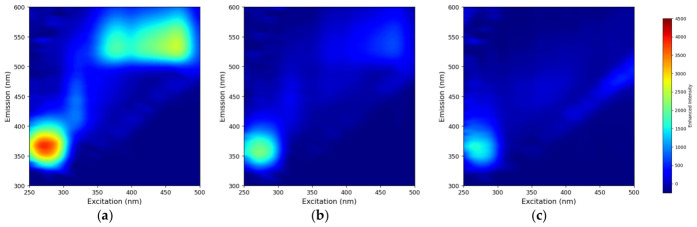
Contour maps for the three-dimensional fluorescence spectra of (**a**) pure bovine milk, (**b**) 50% adulterated milk, and (**c**) pure coconut milk.

The excitation–emission matrix (EEM) fluorescence spectra of pure bovine milk, milk adulterated sample, and pure coconut milk after normalization are presented in Figure 3. Normalization ensured that the spectral patterns across different adulteration levels were comparable and suitable for subsequent multivariate and deep learning analyses [18]. EEM spectroscopy combined with multi-way chemometrics is a robust methodology applicable for the analysis of complex sample matrices [46], making it particularly suitable for characterizing compositionally distinct milk types. Pure bovine milk (S-0) exhibited two prominent fluorescence regions. The first region was observed at excitation/emission wavelengths of approximately 280/340 nm, which is attributed to the intrinsic fluorescence of tryptophan residues present in milk proteins [8,47]. The intrinsic fluorescence of milk proteins originates primarily from three aromatic amino acids (tryptophan, tyrosine, and phenylalanine) [48], dominated by tryptophan due to its high coefficient of extinction [49], and the emission spectra can be linked to protein content and the structural state of milk proteins. Previous several studies have reported the emission fluorescence spectrum of tryptophan at an excitation of 290 nm [50,51,52], with a peak intensity of around 340 nm [53]. The second fluorescence region was detected at excitation/emission wavelengths of approximately 450/520 nm, which corresponds to riboflavin (vitamin B2), a naturally occurring fluorophore in bovine milk.

In the adulterated sample (Figure 3b), the fluorescence intensities of both tryptophan and riboflavin were visibly reduced compared to pure bovine milk, suggesting a dilution effect caused by the addition of coconut milk. Adulteration produces a measurable decrease in riboflavin fluorescence, demonstrating that intrinsic fluorophores in milk can serve as reliable internal biomarkers for detecting adulteration [54]. In contrast, pure coconut milk (Figure 3c) displayed markedly weaker fluorescence across the entire EEM landscape, with both characteristic bovine peaks nearly absent. This observation is consistent with the fact that coconut milk, as a plant-based matrix, lacks the casein-bound tryptophan and riboflavin levels characteristic of bovine milk [55,56]. These results are consistent with previous studies, which reported that fluorescence intensity changes systematically with increasing levels of adulteration [57]. Coconut milk differs fundamentally in its compositional profile compared to bovine milk, and such compositional differences form the basis for spectroscopic discrimination between the two matrices [58]. The visual differences in EEM profiles between pure bovine milk and pure coconut milk, along with the gradual attenuation of fluorescence signals at intermediate adulteration levels, confirm that fluorescence EEM spectroscopy can capture meaningful spectral variations related to coconut milk adulteration, providing a strong foundation for the chemometric models developed in this study.

### 3.2. Parallel Factor Analysis (PARAFAC) Result

The optimal number of PARAFAC components was determined by jointly evaluating the core consistency diagnostic and the explained variance for models ranging from one to five components [59,60], as summarized in Table 1. The number of components is determined based on several criteria; the visual appearance of the loadings is a useful diagnostic because fluorescence spectra of liquids are typically characterized by broad and often unimodal peaks [33], and the variance explained by the model, alongside the core consistency diagnostic, are also considered.

As shown in Table 1, the one-component model produced a low explained variance of 52.0% with a core consistency diagnostic of 100.0%, indicating the model was too simplistic to adequately represent the fluorescence data structure. Extending to two components substantially increased the explained variance to 95.7% while preserving a very high core consistency of 98.7%, signaling a well-structured and chemically valid trilinear model. In contrast, the three-component model caused the core consistency to collapse to 0.0%, with models of four and five components yielding identical results. Therefore, the two-component PARAFAC model was selected as optimal for this dataset.

**Figure 4:**
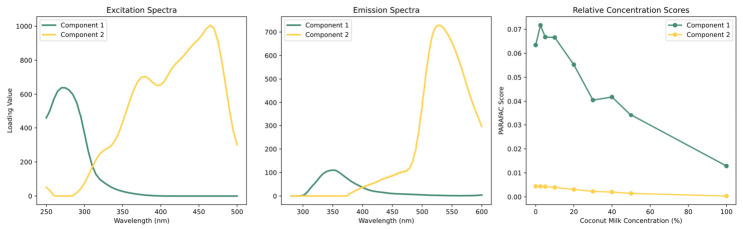
PARAFAC excitation/emission spectra and scores.

The excitation loadings, emission loadings, and relative concentration scores of the two-component model are presented in Figure 4. The obtained scores and loadings from PARAFAC can be directly related to the relative concentration and the fluorescence characteristics of the fluorophores present, meaning that the excitation and emission loadings are used for the interpretation and identification of the resolved fluorescence phenomena. Component 1 exhibited an excitation maximum at approximately 270–290 nm and an emission maximum near 340–350 nm, which is highly characteristic of tryptophan, an intrinsic fluorescent amino acid that dominates the emission of milk proteins. Component 2 displayed a broader excitation band with a peak maximum around 380 nm and 460 nm, paired with an emission maximum at approximately 525 nm. This perfectly matches the well-documented multidimensional fluorescence fingerprint of riboflavin (Vitamin B2), a highly fluorescent photosensitizer naturally occurring in bovine dairy products.

The validity of these chemical assignments is further corroborated by the sample scores. In a trilinear decomposition, the scores act as direct estimates of the relative concentrations of the identified fluorophores. As depicted in the relative concentration plots (Figure 4), the score value for Component 1 (tryptophan) shows a near-linear decrease as the concentration of coconut milk increases from 0% to 100%. A corresponding decrease trend is observed for Component 2 (riboflavin). This behavior aligns perfectly with the biochemical reality of the samples: bovine milk is naturally rich in protein and riboflavin, whereas coconut milk contains negligible amounts of these specific fluorophores. Consequently, the progressive addition of the plant-based adulterant effectively dilutes the bovine milk matrix, leading to a proportional attenuation of the intrinsic tryptophan and riboflavin fluorescence signals.

### 3.3. PLSR Result

The PLSR models were developed using emission spectra extracted at three excitation wavelengths selected based on the dominant fluorophores identified in the PARAFAC analysis: 290 nm (tryptophan) [48,50,61], 330 nm (vitamin A/retinol) [47,62], and 450 nm (riboflavin) [63]. The performance of all 18 model combinations—three excitation wavelengths × three preprocessing methods × two data conditions (non-augmented and augmented)—is summarized in Table 2. The optimum PLSR model is the one for which the coefficient of determination R^2^ is maximum and close to 1, while the error values RMSEP are minimum [64]. Among all models evaluated, those built at the 450 nm excitation wavelength with data augmentation consistently delivered the strongest predictive performance, with the raw-preprocessed model achieving the best overall result (R^2^P = 0.97, RMSEP = 5.00%, RPD = 6.03). This is consistent with the PARAFAC analysis (Figure 4), where Component 2 (identified as riboflavin), showed the most pronounced and monotonic score decline with increasing coconut milk concentration, confirming its role as the most quantitatively reliable spectral biomarker in this dataset.

Compared with the commonly used PLSR model, deep learning approaches resulted in higher prediction accuracy in several adulteration detection studies [17,65]. However, PLSR remains competitive when the input spectral region is carefully selected and the training set is appropriately augmented. The 450 nm augmentation + SG1 model similarly performed well (R^2^P = 0.93, RMSEP = 8.24%), with the lowest LOD of 11.28%, making it preferable when minimizing detection limit is the priority. Models at 290 nm and 330 nm showed generally lower predictive performance, particularly in the non-augmented condition: the 290 nm non-augmented models resulted R^2^P = 0.83–0.92 with RMSEP = 8.61–12.41%, while the 330 nm non-augmented models produced the weakest results compared to all models (R^2^P = 0.78–0.94, RMSEP = 7.64–13.97%). The effect of data augmentation was universally positive, with the most improvements observed at 290 nm (MSC: RMSEP improved from 12.41% to 8.76%) and 450 nm raw (RMSEP: 7.02% → 5.00%), confirming that expanding the diversity of the calibration set substantially enhances model robustness on unseen prediction samples.

### 3.4. 1D-CNN

To evaluate whether a deep learning approach could improve upon PLSR using the same input data, a 1D-CNN was developed using identical emission spectra at the same three excitation wavelengths (290, 330, and 450 nm) with the same 70:30 calibration-prediction split and data augmentation strategy. The predictive performance of the 1D-CNN models across the three excitation wavelengths is summarized in Table 3.

All three models achieved a consistently high R^2^C of 0.99, indicating that the 1D-CNN successfully learned the calibration data structure across all excitation wavelengths. On the independent prediction set, the 450 nm model delivered the best performance (R^2^P = 0.94, RMSEP = 6.75%, RPD = 4.54), followed by 290 nm (R^2^P = 0.92, RMSEP = 8.03%, RPD = 3.82) and 330 nm (R^2^P = 0.91, RMSEP = 8.77%, RPD = 3.49). An RPD value greater than 3 is generally considered the threshold for a good predictive model, and values substantially above this reflect strong practical applicability. All three models exceeded this threshold, though the 450 nm model demonstrated notably superior generalizability, consistent with the dominance of riboflavin fluorescence identified in the PARAFAC analysis.

**Figure 5:**
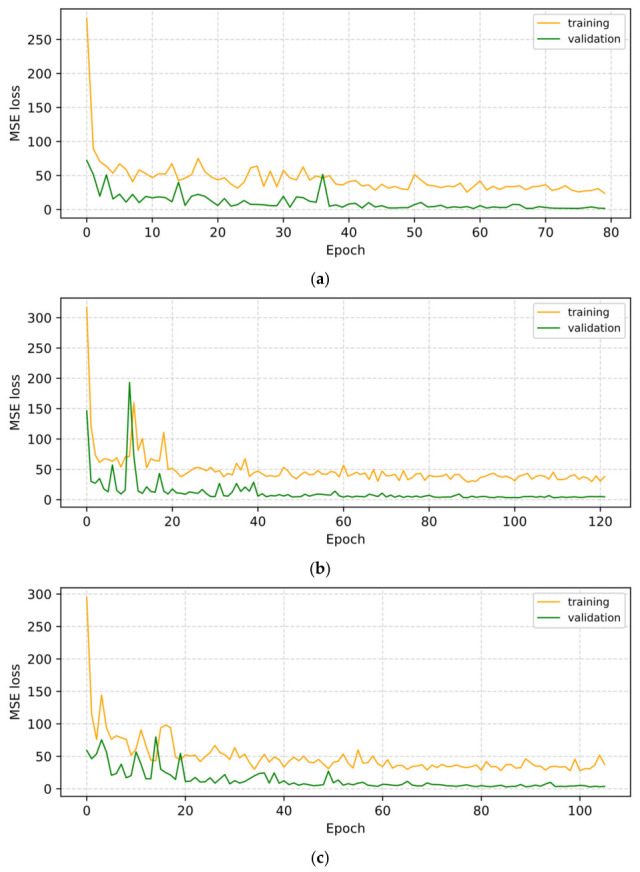
Training and validation loss curve (MSE) of (a) 290; (b) 330; (c) 450 excitation wavelengths.

The training history plots for all three models are presented in Figure 5. In all cases, the training loss remained consistently higher than the validation loss throughout the training process, which is an expected consequence of the data augmentation strategy applied to the calibration set. All three models demonstrated a clear downward trend in both losses, with validation loss converging rapidly and stabilizing at low values, confirming that the models generalized well to unseen prediction samples without overfitting. Early stopping was triggered before the maximum epoch limit in all cases, indicating that optimal model weights were successfully restored prior to performance degradation.

Despite achieving competitive results, both PLSR and 1D-CNN are fundamentally constrained by their reliance on emission spectra at pre-selected discrete excitation wavelengths, meaning the full two-dimensional topographic structure of the EEM contour map is discarded before modeling. This limitation motivates the use of a 2D-CNN that takes the complete EEM image as input, as described in the following section.

### 3.5. 2D-CNN

The 2D-CNN model was evaluated under two data conditions, non-augmented (63 training images) and augmented (630 training images), and the results are summarized in Table 4.

EEM fluorescence spectroscopy coupled with convolutional neural networks has been effectively employed to identify food products and quantify their key components based on distinct fluorescent profiles, demonstrating the strong potential of treating EEM data as two-dimensional spatial images rather than reducing them to one-dimensional spectral vectors. Unlike PLSR and 1D-CNN, which receive only a one-dimensional emission spectrum at a single pre-selected excitation wavelength, the 2D-CNN receives the full EEM contour map as a spatial image input, enabling simultaneous learning from all excitation–emission interactions across the entire fluorescence landscape without any prior wavelength selection.

The comparative framework of this study was specifically designed to demonstrate the benefit of utilizing increasingly richer input representations, rather than to strictly isolate algorithmic effects. Consequently, each model was provided with the input format that best optimized its inherent architectural strengths: PLSR utilized preprocessed 1D spectral vectors, the 1D-CNN required z-score standardized arrays for stable gradient updates, and the 2D-CNN leveraged globally normalized contour map images to extract spatial features. This approach reveals that beyond the choice of the machine learning algorithm itself, preserving the spatial integrity of the EEM data significantly enhances predictive accuracy. While a systematic exploration of various preprocessing combinations for CNN architecture remains a valuable direction for future research, the current findings clearly validate the advantage of utilizing optimized 2D spatial inputs for resolving complex fluorescence landscapes.

This theoretical advantage of using full spatial input was clearly reflected in the model’s quantitative results. Even without augmentation, the 2D-CNN achieved strong predictive performance (R^2^P = 0.97, RMSEP = 4.35%, RPD = 7.05), demonstrating that the full EEM contour map contains sufficient spatial information for accurate quantification of coconut milk adulteration. With augmentation, performance improved substantially, achieving (R^2^P = 0.99, RMSEP = 2.36%, RPD = 12.97, and LOD = 7.80%), representing the best results among all models evaluated in this study. The reduction in RMSEP from 4.35% to 2.36% confirms that augmentation significantly enhanced model generalizability by expanding the diversity of the training set. Deep learning models integrated with spectroscopic technologies have demonstrated superior predictive accuracy in food authenticity tasks, with CNN-based models reaching high coefficients of determination and low prediction errors across a wide range of adulteration detection applications.

**Figure 6:**
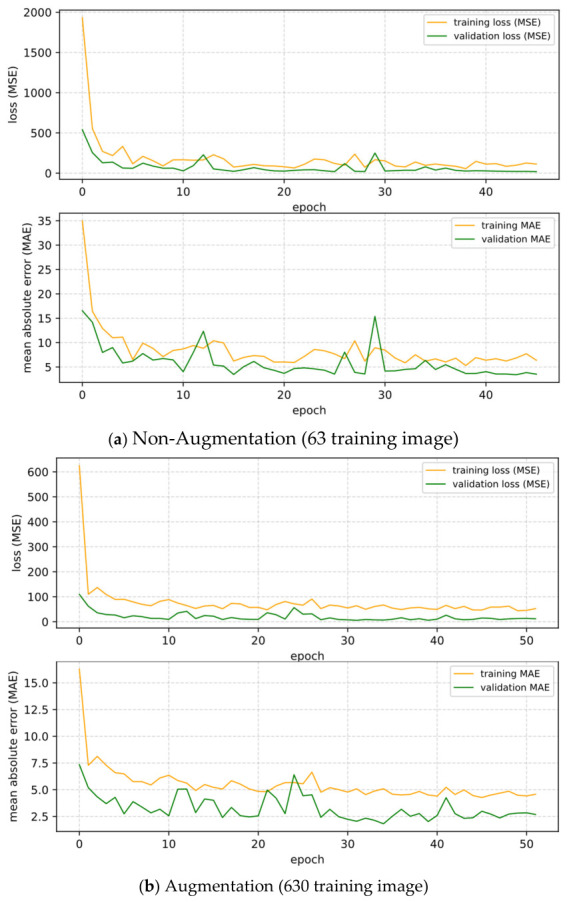
Training and validation learning curves over epoch for (**a**) non-augmented input; (**b**) augmented input.

The training history plots (Figure 6) for both conditions showed that validation loss remained consistently lower than training loss, an expected pattern arising from augmentation being applied to the calibration set. Both MSE and MAE curves converged smoothly in each condition, confirming stable model generalization despite the small original dataset size.

## 4. Conclusions

The superior performance of the 2D-CNN over both PLSR and 1D-CNN is attributed to its fundamentally richer input representation. CNN achieves superior performance compared to classical regression methods by learning hierarchical spatial features directly from raw input without manual feature engineering. By taking the full EEM contour map as a two-dimensional image input, the 2D-CNN simultaneously captures all excitation–emission interactions across the entire fluorescence landscape, including the tryptophan (ex/em: 290/350 nm) and riboflavin (ex/em: 450/525 nm) regions and all spectral interactions between them. This information is inevitably discarded when the data is reduced to a one-dimensional emission slice at a single excitation wavelength as in PLSR and 1D-CNN.

In terms of performance, the 2D-CNN model consistently showed better results than the other approaches, achieving high predictive accuracy with an R^2^P of 0.99, RMSEP of 2.36%, RPD of 12.97, and LOD of 7.80%. This improvement can be attributed to its ability to utilize the full excitation–emission information, allowing more comprehensive feature extraction compared to models that rely on reduced spectral inputs. The PARAFAC analysis further supported these findings by confirming that the observed decrease in fluorescence intensity is associated with the reduction of intrinsic fluorophores, particularly tryptophan and riboflavin, as the proportion of coconut milk increases. Overall, these results highlight the potential of combining EEM fluorescence spectroscopy with deep learning as an accurate and non-destructive approach for detecting milk adulteration in practical applications.

It should be acknowledged that this study was conducted as a proof-of-concept of the proposed EEM-based deep learning framework. The data set was established using only two commercial bovine milk brands and one coconut milk brand, which may not fully capture the natural biological variance of milk arising from differences in animal breed, diet, seasonality, and geographic origin. Furthermore, while PLSR was evaluated across multiple preprocessing methods, the deep learning architectures were assessed using specifically optimized input formats rather than an exhaustive preprocessing comparison. Future research should systematically explore the effects of various mathematical preprocessing techniques on CNN performance, including a wider variety of milk brands to ensure global applicability, as well as the incorporation of other potential adulterants commonly encountered in the dairy supply chain.

## Figures and Tables

**Table 1:** Explained variance as a percentage vs. the number of components for PARAFAC models with one to five components.

	No. of Components				
	1	2	3	4	5
Explained variance (%)	52.0	95.7	98.9	99.1	99.3
Core consistency (%)	100.0	98.7	0.0	0.0	0.0

**Table 2:** Performance of PLSR models at each excitation wavelength.

Wavelength	Preprocessing	R^2^C	RMSEC	R^2^V	RMSEV	R^2^P	RMSEP	RPD	LOD
290 (n-a)	MSC	0.91	9.28	0.82	12.59	0.83	12.41	2.43	41.55
	SG1	0.93	8.11	0.87	10.85	0.90	9.44	3.19	35.83
	Raw	0.91	9.08	0.89	10.10	0.92	8.61	3.50	33.32
290 (a)	MSC	0.98	4.05	0.98	4.27	0.92	8.76	3.44	14.08
	SG1	0.98	3.96	0.97	4.36	0.89	10.19	2.95	14.40
	Raw	0.97	4.87	0.97	5.01	0.88	10.54	2.86	16.53
330 (n-a)	MSC	0.95	6.69	0.94	7.41	0.94	7.64	3.94	24.47
	SG1	0.91	9.17	0.80	13.44	0.78	13.97	2.16	44.37
	Raw	0.90	9.58	0.88	10.58	0.87	10.78	2.79	34.92
330 (a)	MSC	0.99	3.64	0.98	3.83	0.88	10.32	2.92	12.64
	SG1	0.99	3.32	0.98	3.64	0.73	15.74	1.91	12.03
	Raw	0.96	6.17	0.95	6.57	0.84	11.93	2.53	21.69
450 (n-a)	MSC	0.96	5.79	0.93	7.68	0.87	10.66	2.83	25.34
	SG1	0.94	7.23	0.92	8.00	0.92	8.30	3.63	26.43
	Raw	0.96	6.27	0.95	6.91	0.95	7.02	4.29	22.80
450 (a)	MSC	0.99	3.27	0.99	3.35	0.74	15.42	1.95	11.06
	SG1	0.99	3.16	0.98	3.41	0.93	8.24	3.65	11.28
	Raw	0.97	4.78	0.97	4.87	0.97	5.00	6.03	16.09

Processed with data: (n-a) non-augmentation; (a) augmentation. RMSEV: root mean square error of validation; RMSEP: root mean square error of prediction.

**Table 3:** Performance of 1D-CNN models at each excitation wavelength.

Wavelength (nm)	R^2^C	RMSEC	R^2^P	RMSEP	RPD
290	0.99	0.91	0.92	8.03	3.82
330	0.99	2.00	0.91	8.77	3.49
450	0.99	1.52	0.94	6.75	4.54

**Table 4:** Performance of 2D-CNN models with and without augmentation.

	R2C	RMSEC	R2P	RMSEP	RPD	LOD
Non-Augmented	0.97	4.96	0.97	4.35	7.05	14.36
Augmented	0.99	2.08	0.99	2.36	12.97	7.80

## Data Availability

Data will be made available on request.

## References

[1] Haug A., Høstmark A.T., Harstad O.M. (2007). Bovine milk in human nutrition—A review. Lipids Health Dis..

[2] Cimmino F., Catapano A., Petrella L., Villano I., Tudisco R., Cavaliere G. (2023). Role of Milk Micronutrients in Human Health. Front. Biosci.-Landmark.

[3] Johnson R. (2014). Food Fraud and “Economically Motivated Adulteration” of Food and Food Ingredients.

[4] McClements D.J. (2020). Development of Next-Generation Nutritionally Fortified Plant-Based Milk Substitutes: Structural Design Principles. Foods.

[5] Poonia A., Jha A., Sharma R., Singh H.B., Rai A.K., Sharma N. (2017). Detection of adulteration in milk: A review. Int. J. Dairy Technol..

[6] Ullah N., Haseeb A., Tuzen M. (2024). Application of Recently used Green Solvents in Sample Preparation Techniques: A Comprehensive Review of Existing Trends, Challenges, and Future Opportunities. Crit. Rev. Anal. Chem..

[7] Feng Y., Zhang X., Liu J., Yuan Z., Gao S., Shi J. (2025). Advancing food safety and quality assessment: A comprehensive review of non-destructive analytical technologies. Anal. Methods.

[8] Andersen C.M., Mortensen G. (2008). Fluorescence Spectroscopy: A Rapid Tool for Analyzing Dairy Products. J. Agric. Food Chem..

[9] Sádecká J., Tóthová J. (2007). Fluorescence spectroscopy and chemometrics in the food classification—A review. Czech J. Food Sci..

[10] Guimet F., Ferré J., Boqué R., Rius F.X. (2004). Application of unfold principal component analysis and parallel factor analysis to the exploratory analysis of olive oils by means of excitation–emission matrix fluorescence spectroscopy. Anal. Chim. Acta.

[11] Al-Awadhi M.A., Deshmukh R.R. (2021). Detection of Adulteration in Coconut Milk using Infrared Spectroscopy and Machine Learning. Proceedings of the 2021 International Conference of Modern Trends in Information and Communication Technology Industry (MTICTI).

[12] Zheng W., Fu X., Ying Y. (2014). Spectroscopy-based food classification with extreme learning machine. Chemom. Intell. Lab. Syst..

[13] Zhang B., Asad Rahman M., Liu J., Huang J., Yang Q. (2023). Real-time detection and analysis of foodborne pathogens via machine learning based fiber-optic Raman sensor. Measurement.

[14] Liang N., Sun S., Zhang C., He Y., Qiu Z. (2022). Advances in infrared spectroscopy combined with artificial neural network for the authentication and traceability of food. Crit. Rev. Food Sci. Nutr..

[15] Chaudhary V., Kajla P., Dewan A., Pandiselvam R., Socol C.T., Maerescu C.M. (2022). Spectroscopic techniques for authentication of animal origin foods. Front. Nutr..

[16] Huang W., Guo L., Kou W., Zhang D., Hu Z., Chen F., Chu Y., Cheng W. (2022). Identification of adulterated milk powder based on convolutional neural network and laser-induced breakdown spectroscopy. Microchem. J..

[17] Wang L., Liang J., Li F., Guo T., Shi Y., Li F., Hao S., Xu H. (2024). Deep learning based on the Vis-NIR two-dimensional spectroscopy for adulteration identification of beef and mutton. J. Food Compos. Anal..

[18] Lun Z., Wu X., Dong J., Wu B. (2025). Deep Learning-Enhanced Spectroscopic Technologies for Food Quality Assessment: Convergence and Emerging Frontiers. Foods.

[19] Lin H., Li Z., Lu H., Sun S., Chen F., Wei K., Ming D. (2019). Robust Classification of Tea Based on Multi-Channel LED-Induced Fluorescence and a Convolutional Neural Network. Sensors.

[20] Cao S., Yu J., Fan X., Zhang Q., Xue F., Hou D. (2024). Identification of Mixed Organic Pollutants in River Water Based on Graph Convolutional Networks. Proceedings of the 2024 China Automation Congress (CAC).

[21] Sitorus A., Lapcharoensuk R. (2024). Exploring Deep Learning to Predict Coconut Milk Adulteration Using FT-NIR and Micro-NIR Spectroscopy. Sensors.

[22] Zacharioudaki D.-E., Fitilis I., Kotti M. (2022). Review of Fluorescence Spectroscopy in Environmental Quality Applications. Molecules.

[23] Khan M.F.S., Akbar M., Wu J., Xu Z. (2022). A review on fluorescence spectroscopic analysis of water and wastewater. Methods Appl. Fluoresc..

[24] Engelen S., Møller S.F., Hubert M. (2007). Automatically identifying scatter in fluorescence data using robust techniques. Chemom. Intell. Lab. Syst..

[25] Rinnan Å., Andersen C.M. (2005). Handling of first-order Rayleigh scatter in PARAFAC modelling of fluorescence excitation–emission data. Chemom. Intell. Lab. Syst..

[26] Rinnan Å., Booksh K.S., Bro R. (2005). First order Rayleigh scatter as a separate component in the decomposition of fluorescence landscapes. Anal. Chim. Acta.

[27] Bahram M., Bro R., Stedmon C., Afkhami A. (2006). Handling of Rayleigh and Raman scatter for PARAFAC modeling of fluorescence data using interpolation. J. Chemom..

[28] Li X., Zhou Z., Wang X. (2026). Rapid factorization of single EEM for dissolved organic matter analysis. Spectrochim. Acta A Mol. Biomol. Spectrosc..

[29] Mahamuni G., Rutherford J., Davis J., Molnar E., Posner J.D., Seto E., Korshin G., Novosselov I. (2020). Excitation–Emission Matrix Spectroscopy for Analysis of Chemical Composition of Combustion Generated Particulate Matter. Environ. Sci. Technol..

[30] Bro R. (1997). PARAFAC. Tutorial and applications. Chemom. Intell. Lab. Syst..

[31] Hougaard A.B., Lawaetz A.J., Ipsen R.H. (2013). Front face fluorescence spectroscopy and multi-way data analysis for characterization of milk pasteurized using instant infusion. LWT Food Sci. Technol..

[32] Thygesen L.G., Rinnan Å., Barsberg S., Møller J.K.S. (2004). Stabilizing the PARAFAC decomposition of fluorescence spectra by insertion of zeros outside the data area. Chemom. Intell. Lab. Syst..

[33] Andersen C.M., Bro R. (2003). Practical aspects of PARAFAC modeling of fluorescence excitation-emission data. J. Chemom..

[34] Bjerrum E.J., Glahder M., Skov T. (2017). Data Augmentation of Spectral Data for Convolutional Neural Network (CNN) Based Deep Chemometrics. arXiv.

[35] Peleato N.M. (2022). Application of convolutional neural networks for prediction of disinfection by-products. Sci. Rep..

[36] Itakura K., Saito Y., Suzuki T., Kondo N., Hosoi F. (2018). Estimation of Citrus Maturity with Fluorescence Spectroscopy Using Deep Learning. Horticulturae.

[37] Wang X., Wang T., Ji R., Chen H., Qin H., Huang Z. (2025). Identification of Goat Milk Adulterated with Cow Milk Based on Total Synchronous Fluorescence Spectroscopy Combined with CNN. Food Anal. Methods.

[38] An G. (1996). The Effects of Adding Noise During Backpropagation Training on a Generalization Performance. Neural Comput..

[39] Zur R.M., Jiang Y., Pesce L.L., Drukker K. (2009). Noise injection for training artificial neural networks: A comparison with weight decay and early stopping. Med. Phys..

[40] Piotrowski A.P., Rowinski P.M., Napiorkowski J.J. (2012). Comparison of evolutionary computation techniques for noise injected neural network training to estimate longitudinal dispersion coefficients in rivers. Expert Syst. Appl..

[41] Kulko R.-D., Hanus A., Elser B. (2025). Generative Artificial Intelligence for Synthetic Spectral Data Augmentation in Sensor-Based Plastic Recycling. Sensors.

[42] Chen A.-Q., Wu H.-L., Wang T., Wang X.-Z., Sun H.-B., Yu R.-Q. (2023). Intelligent analysis of excitation-emission matrix fluorescence fingerprint to identify and quantify adulteration in camellia oil based on machine learning. Talanta.

[43] Hu Y., Chen X., Li X., Kong D. (2025). Application of excitation-emission fluorescence spectroscopy and 2DCNN for quantitative analysis of diesel emulsified oil content. Anal. Methods.

[44] Lohumi S., Kandpal L.M., Seo Y.W., Cho B.K. (2016). Net Analyte Signal-based Quantitative Determination of Fusel Oil in Korean Alcoholic Beverage Using FT-NIR Spectroscopy. J. Biosyst. Eng..

[45] Cahyarani S.M.D., Aji Nugraha D., Adhitama Putra Hernanda R., Lee H., Zuhrotul Amanah H. (2026). Non-Destructive Moisture Content Prediction Model for Corn Starch Based on Near-Infrared Spectroscopy and Chemometrics. J. Ilm. Rekayasa Pertan. Biosist..

[46] Calvet A., Li B., Ryder A.G. (2012). Rapid quantification of tryptophan and tyrosine in chemically defined cell culture media using fluorescence spectroscopy. J. Pharm. Biomed. Anal..

[47] Ntakatsane M.P., Liu X.M., Zhou P. (2013). Short communication: Rapid detection of milk fat adulteration with vegetable oil by fluorescence spectroscopy. J. Dairy Sci..

[48] Karoui R., Blecker C. (2011). Fluorescence Spectroscopy Measurement for Quality Assessment of Food Systems—A Review. Food Bioprocess Technol..

[49] Ní Fhuaráin Á.M., O’Donnell C.P., Luo J., Gowen A.A. (2024). A Review on MIR, NIR, Fluorescence and Raman Spectroscopy Combined with Chemometric Modeling to Predict the Functional Properties of Raw Bovine Milk. ACS Food Sci. Technol..

[50] Henihan L.E., O’Donnell C.P., Esquerre C., Murphy E.G., O’Callaghan D.J. (2018). Quality Assurance of Model Infant Milk Formula Using a Front-Face Fluorescence Process Analytical Tool. Food Bioprocess Technol..

[51] Ayala N., Zamora A., Rinnan Å., Saldo J., Castillo M. (2020). The effect of heat treatment on the front-face fluorescence spectrum of tryptophan in skim milk. J. Food Compos. Anal..

[52] Birlouez-Aragon I., Sabat P., Gouti N. (2002). A new method for discriminating milk heat treatment. Int. Dairy J..

[53] Freire P., Zamora A., Castillo M. (2024). Synchronous Front-Face Fluorescence Spectra: A Review of Milk Fluorophores. Foods.

[54] Pandey G., Joshi A. (2021). Riboflavin as an internal marker for spoilage and adulteration detection in milk. Food Chem..

[55] Walther B., Guggisberg D., Badertscher R., Egger L., Portmann R., Dubois S., Haldimann M., Kopf-Bolanz K., Rhyn P., Zoller O. (2022). Comparison of nutritional composition between plant-based drinks and cow’s milk. Front. Nutr..

[56] Chalupa-Krebzdak S., Long C.J., Bohrer B.M. (2018). Nutrient density and nutritional value of milk and plant-based milk alternatives. Int. Dairy J..

[57] Ullah R., Khan S., Ali H., Bilal M. (2020). Potentiality of using front face fluorescence spectroscopy for quantitative analysis of cow milk adulteration in buffalo milk. Spectrochim. Acta A Mol. Biomol. Spectrosc..

[58] England P., Tang W., Kostrzewa M., Shahrezaei V., Larrouy-Maumus G. (2020). Discrimination of bovine milk from non-dairy milk by lipids fingerprinting using routine matrix-assisted laser desorption ionization mass spectrometry. Sci. Rep..

[59] Kamstrup-Nielsen M.H., Johnsen L.G., Bro R. (2013). Core consistency diagnostic in PARAFAC2. J. Chemom..

[60] Bro R., Kiers H.A.L. (2003). A new efficient method for determining the number of components in PARAFAC models. J. Chemom..

[61] Shaikh S., O’Donnell C. (2017). Applications of fluorescence spectroscopy in dairy processing: A review. Curr. Opin. Food Sci..

[62] Brandao M.P., Neto M.G., De Carvalho Dos Anjos V., Bell M.J.V. (2017). Detection of adulteration of goat milk powder with bovine milk powder by front-face and time resolved fluorescence. Food Control.

[63] Yang H., Xiao X., Zhao X.S., Hu L., Xue X.F., Ye J.S. (2016). Study on Fluorescence Spectra of Thiamine and Riboflavin. MATEC Web Conf..

[64] Zhao N., Wu Z., Zhang Q., Shi X., Ma Q., Qiao Y. (2015). Optimization of Parameter Selection for Partial Least Squares Model Development. Sci. Rep..

[65] Nallan Chakravartula S.S., Moscetti R., Bedini G., Nardella M., Massantini R. (2022). Use of convolutional neural network (CNN) combined with FT-NIR spectroscopy to predict food adulteration: A case study on coffee. Food Control.

